# Evolution of a New Testis-Specific Functional Promoter Within the Highly Conserved *Map2k7* Gene of the Mouse

**DOI:** 10.3389/fgene.2021.812139

**Published:** 2022-01-05

**Authors:** Tobias Heinen, Chen Xie, Maryam Keshavarz, Dominik Stappert, Sven Künzel, Diethard Tautz

**Affiliations:** ^1^ Uniklinik Köln, Köln, Germany; ^2^ Max-Plank Institute for Evolutionary Biology, Plön, Germany; ^3^ Deutsches Zentrum für Neurodegenerative Erkrankungen e. V. (DZNE), Bonn, Germany

**Keywords:** regulatory evolution, new promotor, kinase, testis, sperm maturation

## Abstract

*Map2k7* (synonym *Mkk7*) is a conserved regulatory kinase gene and a central component of the JNK signaling cascade with key functions during cellular differentiation. It shows complex transcription patterns, and different transcript isoforms are known in the mouse (*Mus musculus*). We have previously identified a newly evolved testis-specific transcript for the *Map2k7* gene in the subspecies *M. m. domesticus*. Here, we identify the new promoter that drives this transcript and find that it codes for an open reading frame (ORF) of 50 amino acids. The new promoter was gained in the stem lineage of closely related mouse species but was secondarily lost in the subspecies *M. m. musculus* and *M. m. castaneus*. A single mutation can be correlated with its transcriptional activity in *M. m. domesticus*, and cell culture assays demonstrate the capability of this mutation to drive expression. A mouse knockout line in which the promoter region of the new transcript is deleted reveals a functional contribution of the newly evolved promoter to sperm motility and the spermatid transcriptome. Our data show that a new functional transcript (and possibly protein) can evolve within an otherwise highly conserved gene, supporting the notion of regulatory changes contributing to the emergence of evolutionary novelties.

## Introduction

Mitogen activated protein kinase (MAPK) pathways are highly conserved throughout eukaryotes and trigger multistep signaling cascades mediating transcriptional response upon receiving outside stimuli (English et al., 1999; Chang and Karin, 2001). *Map2k7* belongs to the JNK group of kinases and acts as its specific activator (Holland et al., 1997; Tournier et al., 1997; Fleming et al., 2000; Tournier et al., 2001; Kishimoto et al., 2003; Takekawa et al., 2005; Wang et al., 2007). Cellular stresses like UV and gamma irradiation, osmotic shock and drug treatments on the one hand and different inflammatory cytokines, such as tumor necrosis factor, interleukin-1 or interleukin-3 on the other hand, lead to JNK pathway activation (Moriguchi et al., 1997; Foltz et al., 1998; Chang and Karin, 2001; Nishina et al., 2004). Downstream targets of JNK include transcription factors (Yang et al., 2003) as well as other proteins, for example microtubule-associated proteins (Chang et al., 2003) and members of the *Bcl2* family (Lei et al., 2002; Deng et al., 2003). The JNK pathway has several known functions in the immune system, apoptosis, and developmental processes (Sabapathy et al., 1999; Dong et al., 2000; Nishina et al., 2004; Wada et al., 2004; Wang et al., 2007). Knock out of both JNK1 and JNK2 (double KO) or of Mapk2k7 alone, leads to embryonic lethality in mice (Wang et al., 2007).

In their report on the first identification of *Map2k7* in mice, Tournier and others (Tournier et al., 1997) showed by Northern blotting the expression of a long transcript in various organs, plus an additional shorter transcript specific to the testis. While all the further studies concentrated on the longer transcript, the origin and function of the shorter transcript was not further investigated. In a systematic study for differentially expressed genes in mouse populations, we identified *Map2k7* to be differentially expressed in testis between wild populations of *M. m. domesticus* and *M. m. musculus*, with strongly elevated expression in *M. m. domesticus* (Harr et al., 2006). A *cis*-trans test via allele-specific expression analysis in F1 hybrids of both subspecies demonstrated that a *cis*-acting sequence causes the expression change. It turned out that the elevated expression level can be correlated with the additional testis-specific transcript in the *M. m. domesticus* subspecies, which is absent in *M. m. musculus*. This suggested that a new testis-specific promoter had evolved within the *Map2k7* gene (Harr et al., 2006). Given the highly conserved nature of the *Map2k7* gene, such an evolution of a strong new promoter is of particular interest. We present here comparative and functional data that allow inferences on the evolutionary history of the new promoter, which includes both a new origination event and a secondary loss event triggered by a single mutation in one of the subspecies. The knockout analysis proves that the new promoter has assumed a new function in spermatids’ maturation and the transcriptome’s regulation during this phase.

## Materials and Methods

### *In situ* Hybridization and Northern Blotting

*In situ* detection of *Map2k7* RNA was performed by hybridization with a digoxigenin (DIG) labeled probe (Tautz and Pfeifle, 1989). For probe generation, a fragment spanning 1197bp of *Map2k7* exons 5-10 was amplified from testis C57Bl6 cDNA with primers P49 and P50 and cloned into a PCR cloning vector. The DNA fragment was reamplified from a pure plasmid clone. Reverse transcription to generate a DIG labeled probe was set up by adding 200 ng of purified PCR product to 2 μl DIG RNA Labeling Mix, 2 μl transcription buffer, 2 μl T7 polymerase, 0.5 μl RNase inhibitor (Roche, Basel). Pure water was added to the reaction mix to obtain a final volume of 20 μl. The reaction mix was incubated for 2 h at 37°C followed by a treatment with 1 μl Turbo DNAse for 15 min at 37°C to remove the DNA template. The probe was precipitated with salt and alcohol, washed and re-suspended in 40 μl of 50% formamide diluted in nuclease free water (Applied Biosystems/Ambion, Austin).

All buffers and tools that were used for the following procedure were kept RNAse free. Paraffinized sections were dewaxed in xylene for 2 × 10 min, washed for 5 min in ethanol, rehydrated in a series of decreasing ethanol concentration (95%, 90%, 70%, 30%; 3 min each) and washed for 5 min in PBS before postfixing them for 1 h in 4% PFA. After postfixation the tissue was washed in PBS for 2 × 5 min and partially digested with 10 μg/ml proteinase K in 100 mM Tris-HCl pH 7.5 for 10 min at 37°C.

Digestion was stopped with 0.2% glycine in PBS. 2 × 5 min washing in PBS was followed by 15 min incubation in 0.1 N HCl and another 2 × 5 min washing in PBS was performed previous to blocking of positively charged amino acids by 0.25% acetic anhydride in 0.1 M triethanolamine pH 8.0 for 10 min. Afterwards slides were washed for 5 min in PBS and for 5 min in pure water before prehybridization for 2 h at 65°C (50% formamide, 5x SSC, 1x Denhardt’s, 0.1% Tween-20). 1 μl of DIG labeled probe was diluted in 100 μl prehybridization buffer containing 400 ng tRNA (Sigma-Aldrich, St. Louis) and denatured at 70°C for 5 min. The hybridization mix was applied to the sections and covered with coverslips. Slides were incubated overnight at 65°C in a moist chamber. The next day, the sections were washed in 50% formamide containing 5x SSC and 1% SDS at 70°C for 30 min and subsequently with 50% formamide containing 2x SSC and 0.2% SDS for another 30 min at 65°C. Afterwards the sections were washed for 3 × 5 min in MABS (100 mM maleic acid, 150 mM NaCl, 0.1% Tween-20, and 2 mM levamisole; adjusted to pH 7.5 with NaOH). Samples were blocked with 1% blocking reagent (Roche, Basel) in MABS. Anti-DIG-AP antibody was applied in 1% blocking reagent in MABS by overnight incubation at 4°C. The next day, the sections were first washed 3 × 10 min and then 3 × 30 min in MABS. Subsequently, pH was adjusted by incubating for 3 × 10 min in NTMLT buffer (100 mM Tris-HCl pH 9.5, 50 mM MgCl_2_, 100 mM NaCl, 100 mM levamisole, 0.1% Tween-20). BM purple solution (Roche, Basel) was applied as a substrate for the alkaline phosphatase coupled with the anti-DIG antibody. The tissue was stained until the desired degree of the signal was observed. Slides were washed for 1 min in water and mounted with Kaisers glycerin-gelatine.

Detection of RNA in Northern Blotting (Alwine et al., 1977) was performed with radioactively labeled probes generated from the same clone used for *in situ* hybridization. Probes were labeled with ^32^P-dCTP (Hartmann Analytic, Braunschweig) using the Rediprime II DNA Labeling Kit (GE Healthcare Life Science, Little Chalfont) according to the manufacturer’s manual. Labeled probes were cleaned up with MicoSpin S-200 HR columns (GE Healthcare Life Science, Little Chalfont) according to the manufacturer’s manual.

10 μg of total RNA per sample were diluted in 15 μl nuclease-free pure water (Applied Biosystems/Ambion, Austin) and mixed with 10 μl sample buffer (50% formamide, 5.18% formaldehyde, 2.5x MOPS, 0.1 mg/ml ethidium bromide and 2.5x blue marker). Samples were heat-denatured for 5 min at 70°C and separated on an agarose gel (1.2% agarose, 0.666% formaldehyde, 1x MOPS). The RNA lanes were blotted through a classic upward blot onto an Amersham Hybond N+ membrane (GE Healthcare Life Science, Little Chalfont) by neutral transfer (20x SSC) overnight. Membranes were baked for 2 h at 80°C and prehybridized in ExpressHyb (Clontech, Mountain View) at 65°C for 1 h. A radioactively labeled probe was added to the prehybridized blot and hybridization took place overnight at 65°C in a rotating oven. The next day, the blots were washed 10–40 min in 2x SSC containing 0.05% SDS at room temperature and subsequently washed for 5–30 min with 0.1x SSC containing 0.1% SDS at 50°C. After washing, the blots were dipped in 2x SSC, sealed in a plastic bag, and analyzed via autoradiography using Kodak Biomax-MS films (Kodak, Rochester).

### Promoter Tests in Cell Culture

The promoter tests in cell culture did require to set up an appropriate expression system. The details on the construction and testing of this system are described in (Heinen, 2008). It resulted in the construction of a “Luciflip plasmid” that contains the following elements in the given order: PGK-promoter, ATG, FRT, splice acceptor, double polyA signal, EcoRI site, Kozak, Luc-MYC, intron, polyA signal and was the basis for the Map2k7 alpha reporter assay (compare Supplementary Figure S1 for further details on this plasmid). For this, fragments spanning −487 to +43 relative to the transcription start of the *Map2k7* alpha promoter (see annotation of the sequence in Supplementary Figure S2) were amplified from genomic DNA of *M. m. musculus* and *M. m. domesticus* using the primer pair P318/P319. A two-step PCR strategy was pursued to generate fragments with a deleted insulator motif. Two separate PCRs with the primer pairs P318/P320 and P321/P319 were run on top of the cloned promoter fragment. The primers P319 and P320 bind just right upstream and downstream of the insulator sequence and were tailed with a sequence stretch that is homologous to the sequence on the opposite part exactly beyond the insulator. The other primer is one of the primers that were used in the first PCR. Thus, the promoter fragment is divided into two fragments, each defined by an inner and an outer primer. The inner edges overlapped but lacked the insulator. Both PCR products were cleaned up and included in another PCR without primers. After five cycles, the outer primers P318 and P319 were added to the reaction, and PCR continued as usual. The resulting product was cloned into a PCR cloning vector and validated by Sanger sequencing with M13 primers.

The second version of all four fragments has an additional upstream CMV enhancer. For this, the CMV enhancer was amplified with the primers P322 and P323 using the phrGFPII-1 plasmid (Agilent Technologies, Santa Clara) as a template. The resulting product was cloned into a PCR cloning vector and validated by Sanger sequencing with M13 primers. Both CMV primers and the upstream primer used for promoter fragment generation (P318) were tailed with an XhoI restriction site overhang. CMV enhancer fragments were retrieved by XhoI digestion and ligated into the XhoI site of all 4 Map2k7 promoter fragments. The assembled fragments were cut out by EcoRI digestion and cloned into the EcoRI site of Luciflip. The orientation of the inserts was controlled with an XhoI digest. All 8 Luci-flip constructs were sequenced with the primers P293, P358, and P199 to control the inserts. No mutations were found.

The 8 expression constructs were transfected into NIH/3T3 cells. For this, NIH/3T3 fibroblast cells were grown in DMEM medium containing sodium pyruvate, non-essential amino acids, L-glutamine penicillin/streptomycin (all from Invitrogen, Carlsbad) and 10% fetal calf serum (PAN, Aidenbach) at 37°C incubation maintaining 5% CO_2_ concentration. One day before transfection, 3 × 10^3^ NIH/3T3 cells were seeded with 70 μl medium into each well of 96-well plates and grown overnight. Cells were co-transfected with Luciflip plasmid and a pGL3 plasmid (Promega, Mannheim) which contains firefly luciferase under the control of SV40 promoter. Firefly luciferase was used to normalize transfection efficiency. Therefore, 0.18 μl Fugene 6 reagent (Roche, Basel) was added to 4.82 μl serum-free medium and incubated for 5 min at room temperature. Subsequently, 30 ng of Luciflip and 30 ng of pGL3 DNA were mixed and added to the medium containing Fugene 6. The mixture was incubated for 20 min and added to one well of the 96 well plate containing NIH/3T3. The transfection was performed for the Luciflip-CMV construct and for an empty Luciflip plasmid as blank control. Each transfection was performed in 8 replicates in parallel. Cells were incubated overnight. The next day, firefly and renilla luciferase substrates were applied using the Dual-Glo Luciferase Assay System (Promega, Mannheim) according to the manufacturer’s manual (the corresponding reagent was added directly to the cells and induced cell lysis). Relative light units were measured with a Mithras LB 940 Luminometer (Berthold Technologies, Bad Wildbad). The renilla luciferase signal was divided by the firefly luciferase signal to normalize transfection efficiency for every well. For every construct, median and standard deviation were calculated from the 8 individual replicates.

### Construction of Knockout Mice

The general scheme for the construction of the promoter knockout is shown in Supplementary Figure S2. It consisted of two steps. In the first step, in neomycin cassette was inserted at position chr8:4,239,573 (mm10) via homologous recombination in embryonic stem cells, whereby 593 bp of the promoter region, including all possible transcription start sites were deleted (compare sequence annotation in Supplementary Figure S2). These cells were then used to generate transgenic mice via injection into blastocysts. In the second step, the neomycin cassette was removed via flp recombination at the FRT sites in the mice. The annotated wildtype sequence in this region, the neomycin cassette, and the final sequence after recombination are provided in Supplementary Figure S2. The generation of the KO mice was done by inGenious Targeting Laboratory (iTL), Stony Brook. The mice were then transferred into our facility and backcrossed against C57Bl6/J until final analysis after about 15 generations.

### Sperm Analysis

Computer-assisted sperm analysis (CASA) using a CEROS Sperm Analyzer (Hamilton Thorne, Beverly MA) was used to study sperm motility, following the protocols described in (Goossens et al., 2008; Turner et al., 2012). Testis and epididymis were dissected from the right side of each mouse, and weights of the testis and the mice were determined to normalize testis weight with body weight. The cauda epididymis was excised, immediately transferred in 250 μl human tubular fluid medium (Millipore, Billerica), punctured with a needle, and placed at 37°C, 5% CO_2_ for 20 min. The sperm suspension was diluted to ∼1 million/ml and ∼25 μl loaded into 100 μm depth slide chambers (Leja, Nieuw-Vennep, Netherlands). The fractions of motile sperm and progressive sperm were calculated by the CEROS system [see (Turner et al., 2012) for a discussion of the different motility parameters in mice].

### RNA-Seq and Data Analysis

The testis tissues of 8 WT and 8 knockout mice were carefully collected and immediately frozen in liquid nitrogen. Total RNA was purified using QIAGEN RNeasy Microarray Tissue Mini Kit (Catalog no. 73304), and prepared using Illumina TruSeq Stranded mRNA HT Library Prep Kit (Catalog no. RS-122-2103), and sequenced using Illumina NovaSeq 6000 Regaent Kit v1.5 (200 cycles) (Catalog no. 20028313). Raw reads in FASTQ format were trimmed with Trimmomatic (0.38) (Bolger et al., 2014), and only the reads left in pairs were used for further analysis. The trimmed reads were mapped to the mouse reference genome GRCm39 (Waterston et al., 2002; Howe et al., 2021) with HISAT2 (2.2.1) (Kim et al., 2015) and SAMtools (1.9) (Li et al., 2009), and the mouse gene annotation in Ensembl (Version 104) was used for indexing the genome, i.e., the options “--ss” and “--exon” were used for command “hisat2-build”. The numbers of fragments uniquely mapped to the genes annotated in Ensembl (Version 104) were calculated with featureCounts (2.0.3) (Liao et al., 2014). Principal component analysis on VST (varianceStabilizingTransformation) fragment counts and differential expression analysis on raw counts were performed with DESeq2 (1.30.1) (Love et al., 2014; Zhu et al., 2019).

### Primers Used

All primers were obtained from Metabion (Martinsried). Sequences are listed 5′> 3′.

P49 AAT​TAA​CCC​TCA​CTA​AAG​GGGAGC​ATC​GAG​ATT​GAC​CAG​A

P50 TAA​TAC​GAC​TCA​CTA​TAG​GGGCT​CGG​ATG​TCA​TAG​TCA​GG

(Underlined parts in P49 and P50 indicate the matches with the mouse genomic sequence).

P318 CTC​GAG​TGA​CCA​ACT​ACT​TTT​CAC​TAT​TGC​TG

P319 CAA​GCT​GTG​AAG​GTC​AGT​CAG​G

P320 TGG​TGG​ACA​AGC​TGG​ATC​TAG​AAA​GGA​AGA​GGA​AGC​ACT

P321 CTC​TTC​CTT​TCT​AGA​TCC​AGC​TTG​TCC​ACC​ATG​ACC

P322 CTC​GAG​CGC​GTT​ACA​TAA​CTT​ACG​GTA​AA

P323 CTC​GAG​CAA​AAC​AAA​CTC​CCA​TTG​ACG

## Results

### Expression Differences Between Sub-Species

**FIGURE 1:**
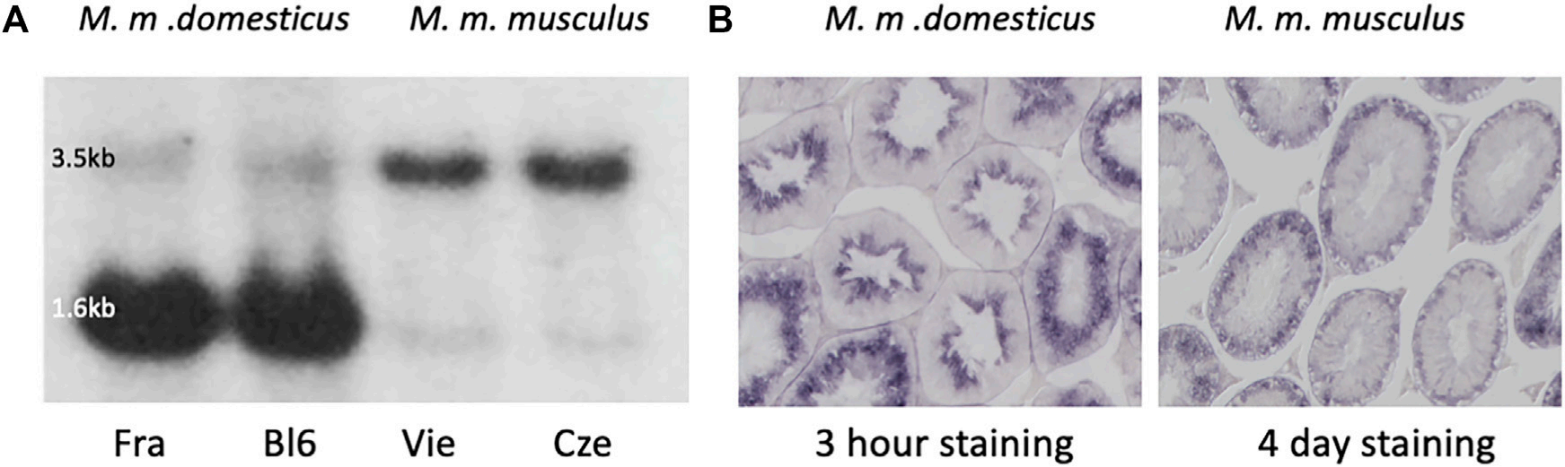
*Map2k7* expression in *M. m. domesticus* versus *M. m. musculus*. **(A)** Northern blots with testis RNA from *M. m. domesticus* individuals of a wild population from France (Fra) and the laboratory inbred strain C57Bl6/J (Bl6), as well as *M. m. musculus* individuals from a wild-caught population from Vienna (Vie) and a wild-caught population from the Czech Republic (Cze). A fragment spanning exons 5-10 was used as a probe (see Methods for generating this fragment). The strongly expressed 1.6 kb band is only visible in the two individuals from the *M. m. domesticus* subspecies. The weak 1.6 kb band in *M. m. musculus* is derived from another transcript variant that differs with respect to an additional intron in the 3′-end of the long transcript (compare annotation in Figure 2). **(B)** *In situ* hybridization with the *Map2k7* probe on cross-sections of seminiferous tubules of an individual from *M. m. domesticus* **(left)** and an individual from *M. m. musculus* **(right)** was the same fragment as used for the Northern blots. Note that the *M. m. domesticus* signal developed already after 3 h of color incubation, while the *M. m. musculus* signal developed only after 4 days of color incubation. This is compatible with the fact that the signal in *M. m. domesticus* comes mainly from the strong 1.6 kb transcript seen in the Northern blot, while the signal in *M. m. musculus* comes from the weaker 3.5 kb transcript. Microscope pictures were taken at ×100 magnification.

Comparison of testis expression of *Map2k7* in two mouse subspecies (*Mus musculus domesticus*, and *Mus musculus*) via Northern blotting and *in situ* hybridization shows a major difference between the two subspecies (Figure 1). We included in our analysis testis samples from the inbred strain C57Bl6/J [derived from *M. m. domesticus* (Frazer et al., 2007)], as well as from wild-caught mice that were kept under outbreeding conditions (Harr et al., 2016). Northern blotting was done with a 1.2 kb probe spanning exons 5–10 shared between all RNA isoforms (see Methods for the generation of this probe). They revealed a weak 3.5 kb and a strong 1.6 kb band in *M. m. domesticus* (Figure 1A), but only the 3.5 kb band in *M. m. musculus*, with the major 1.6 kb band missing. For the inbred strain C57Bl6/J this confirms the previous observations by (Tournier et al., 1997) and for the wildtype strains the observation by (Harr et al., 2006).

To assess the stage of spermatid development at which *Map2k7* is expressed, we used *in situ* hybridization on testis sections from *M. m. domesticus* and *M. m. musculus*. Testis tissue mainly consists of seminiferous tubules, which are the location of spermatogenesis. Spermatogonial stem cells adjacent to the inner tubule wall divide and form spermatocytes which undergo meiosis. After meiosis, the spermatocytes develop into spermatids and change morphologically from round to elongated spermatids before the generation of mature spermatozoa is completed. The three main stages, spermatogonia, spermatocytes, and spermatids, are classified into further substages (Russell et al., 1990). Sperm precursor cells are embedded in Sertoli cells, which define the shape of the spermatogenic epithelium and support the germ cells. Through the influence of Sertoli cells, developing sperm precursor cells proceed towards the lumen of seminiferous tubules according to their degree of maturation. Terminal spermiation releases the sperm cells into the luminar fluid of the tubules that transfers them to the epididymis. Hence, ring-shaped zones representing different cell stages can be distinguished in a transverse section of seminiferous tubules.

*In situ* hybridization results on testis sections using the same probe as used for the Northern blotting are shown in Figure 1B. In *M. m. domesticus*, we found a strong signal in post-meiotic spermatid stages. In contrast, the *Map2k7* expression pattern in *M. m. musculus* is very weak and becomes only visible after several days of color development. This signal is restricted to pre-meiotic stages, and we interpret it as the expression of the long transcript. This expression would be expected to be present also in *M. m. domesticus*, but the sections are over-stained after several days of incubation, making it impossible to visualize this directly.

### Transcript Structures

**FIGURE 2:**
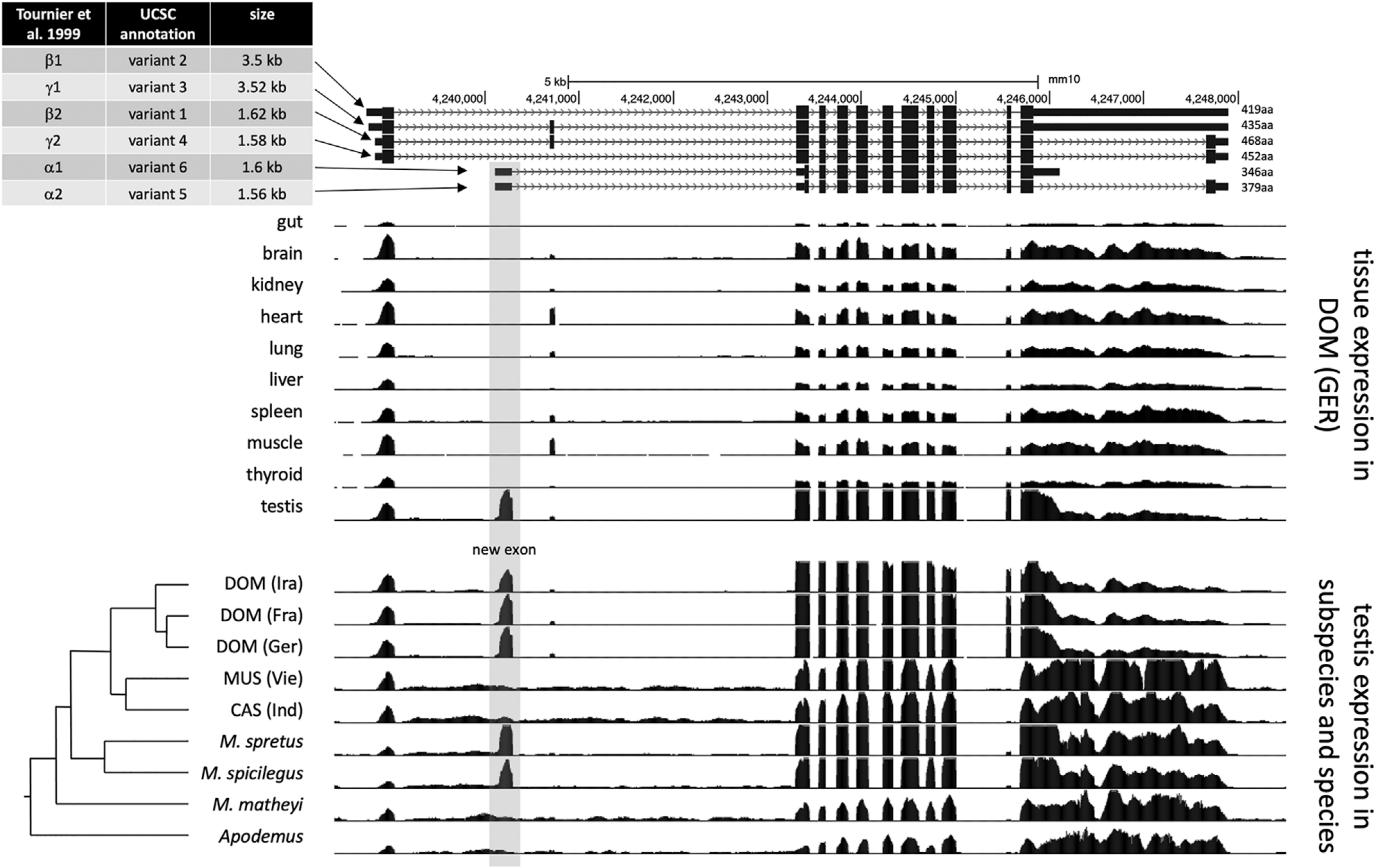
*Map2k7* transcript variants and transcriptome read coverage. The figure is based on UCSC genome browser (Kent et al., 2002) tracks for the mouse GRCm38/mm10 reference sequence (Waterston et al., 2002). The top panel shows the different transcript and splice versions from the mouse reference genome, representing *M. m. domesticus* and therefore includes the new promoter/exon (highlighted by grey shading). The middle panel is based on data from (Harr et al., 2016) and shows transcriptome read mapping tracks for different tissues. The lower panel is based on transcriptome data from (Harr et al., 2016) and (Neme and Tautz, 2016) and shows transcriptome read mapping tracks for different populations, subspecies, and species. The phylogenetic relationships are depicted to the left. DOM: *M. m. domestics*, MUS: *M. m. musculus*, CAS: *M. m. castaneus*.

A nomenclature for the different isoforms of *Map2k7* was introduced by (Tournier et al., 1999) based on the analysis of various cDNA clones. They distinguish α, β, and γ isoforms that have different 5′-exons, which can be alternatively combined with two 3′-exon variants (named 1 and 2 isoforms). However, with the availability of more genome data, their analysis needed to be further updated (for example, in their γ isoform, the exons 2 and 3 should be in reverse order). In the current annotation of the UCSC genome browser, annotators have introduced a new nomenclature by just numbering the isoforms as “variants 1–6” (see Figure 2—top panel). They fall into two major length classes, variants 2 and 3 [nominally γ in (Tournier et al., 1999)] are about 3.5 kb in length and differ only by the presence/absence of a small exon. Variants 1 and 4 [nominally β in (Tournier et al., 1999)] also differ by the presence/absence of this small exon, but have an additional intron in the 3′-exon, which shortens them to about 1.6 kb. Variants 5 and 6 [nominally α in (Tournier et al., 1999)] also have a length of about 1.6 kb, but start with a different 5′-exon and differ at the 3′-end (see Figure 2—top panel).

To better resolve the transcript structures and assess the origin of the new exon, we used the transcriptome data described in (Harr et al., 2016). Figure 2 shows the read coverage as browser tracks aligned to the annotated versions of the *Map2k7* transcripts in the UCSC browser (Figure 2—top panel). The set of browser tracks representing the transcriptomes from 10 different tissues of individuals from a *M. m. domesticus* population show that the new exon (highlighted in grey) is only expressed in the testis (Figure 2—middle panel). The second set of browser tracks shows testis transcriptomes from different populations of the subspecies and closely related species. The new exon is only present in *M. m. domesticus* populations (DOM), as well as in the sister species *M. spretus* and *M. spicilegus*, but absent in the subspecies *M. m. musculus*, *M. m. castaneus*, as well as the further distant mouse species *M. matheyi* and the wood mouse *Apodemus* (Figure 2—lower panel).

Northern blotting and qPCR using different exon-specific probes and primers (Supplementary Figure S3) lead us to conclude that the highly expressed testis-specific ∼1.6 kb fragment corresponds to variant 6 in the UCSC annotation and to Mkk7-α1 in (Tournier et al., 1999). In the following, we will call it Map2k7-α1 to account for the change in the official gene nomenclature.

### Protein Coding Potential

**FIGURE 3:**
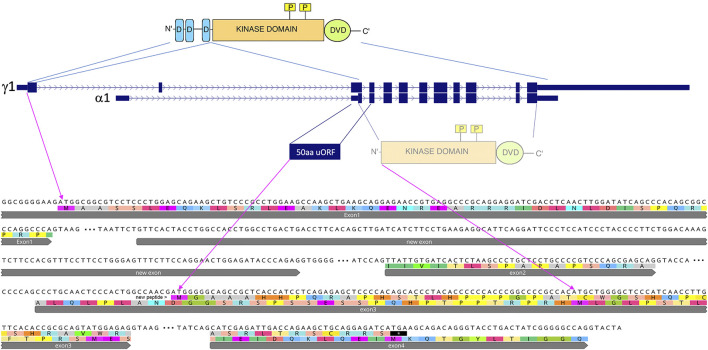
Comparison of the main *Map2k7* transcripts and their coding potentials. Transcript depictions are taken from the UCSC browser annotations (see also Figure 2), whereby only the two main transcripts are shown, the conserved one (γ1) and the new one starting from the new promoter (α1). The protein domains of the *Map2k7* functional kinase are depicted on the top, including the JNK-binding sites (D-domains in blue), the kinase domain (in orange) with its functional phosphorylation sites (yellow), as well as the C-terminal DVD domain (green). The first AUG in the new transcript is in the third exon, and it has a 50aa ORF, which would represent a *de novo* generated protein. If this AUG would not be used, the next AUG in a different frame could potentially lead to the expression of a truncated version of the *Map2k7* protein, containing only the kinase and the DVD domains. The corresponding coding exon sequences (up to exon 4 for the γ1 transcript) are depicted below (flanked by their splice sites, introns are depicted as three dots) with the translations in the two alternative reading frames.

The transcripts Map2k7-β and Map2k7-γ, include the JNK-binding site (D-domains) in the first exon (Figure 3). They are about ∼3.5 kb in size and can be found in all analyzed populations and species. The newly evolved transcription start is situated within the first intron of the conserved transcripts. Its transcript does not include the exon with the D-domains but encodes potentially a protein with the kinase and DVD domain. (Tournier et al., 1999). have shown that this truncated protein has a detectable, but very weak kinase activity when expressed from an expression vector in cell culture. However, the first AUG in the new transcript is before this long ORF and in a different reading frame. It initiates a novel 50aa ORF (Figure 3), and the nucleotides surrounding the start codon of this new ORF (UGGCCAACG AUG G) match much better to the Kozak-consensus-sequence (Kozak, 1987) than the nucleotides surrounding the start codon of the remaining reading frame of the Map2k7-α1 transcript (CCCCGCCAC AUG C). A purine at position −3 and a guanine at position +4 are the most critical sequence elements for translation initiation. It is therefore questionable whether the shortened form of *Map2k7* is translated at all under natural conditions.

Based on the phylogenetic tree of wild mice (Guenet and Bonhomme, 2003; Chevret et al., 2005), we can infer that the new 1.6 kb transcript has arisen before the branching of *M. spicilegus* and *M. spretus* at least 2 million years ago, but not more than 6 million years ago, since it is not present in the outgroups *M. matheyi* and *Apodemus* (Figure 2). Note that while these outgroups show some RNAseq reads mapping to this region, thus indicating general low-level transcription, none of these reads are spliced at the exon-intron boundary seen in the other species. The new promoter activity has secondarily disappeared in the lineage of *M. m. musculus* and *M. m. castaneus*, apparently because of a crucial T/A substitution (see below).

### Promoter Analysis

**FIGURE 4:**
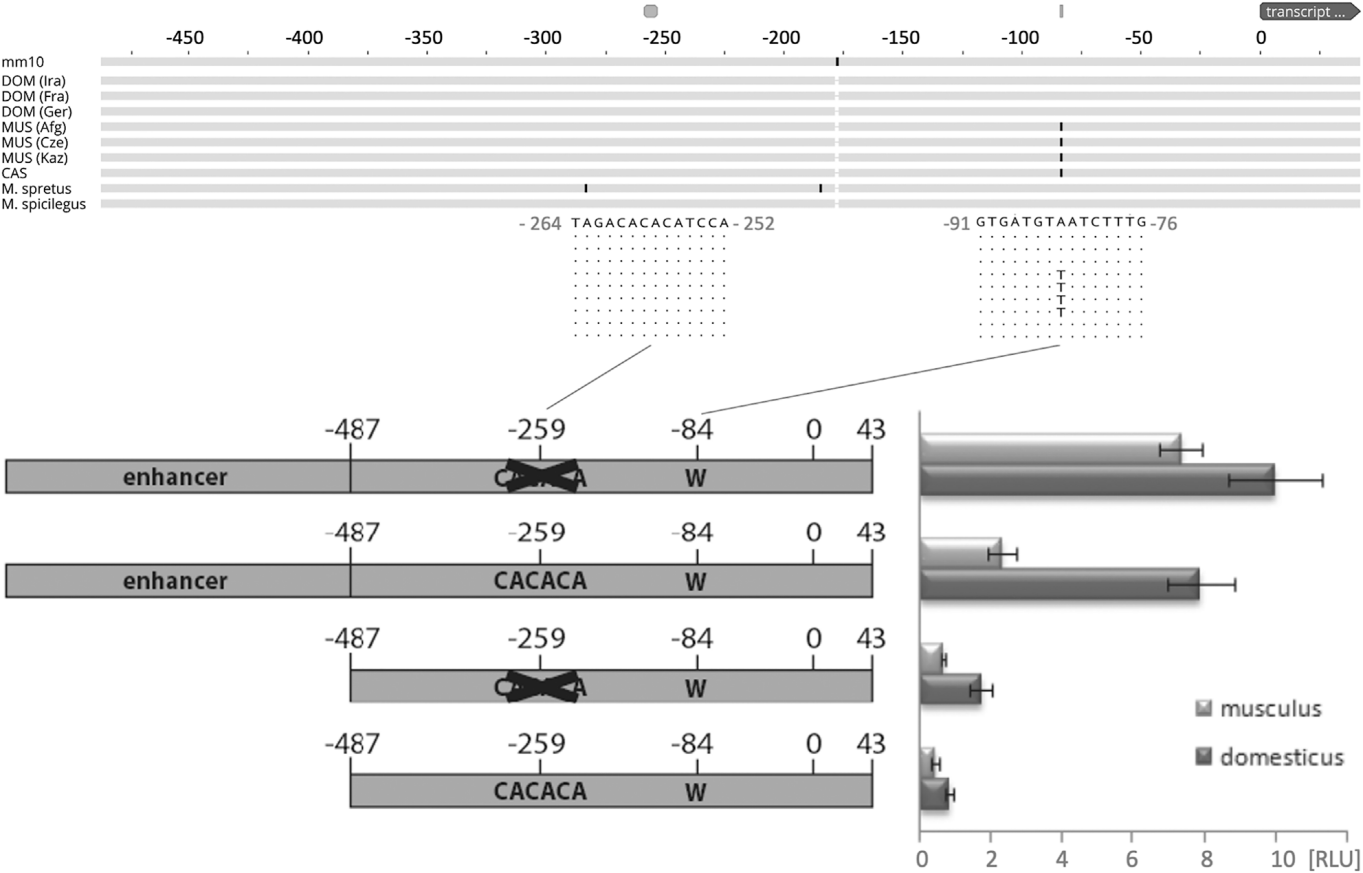
Functional test of the promoter region driving the new transcript. The top shows the alignment scheme of genomic sequences from populations, sub-species, and species based on the genome data described in (Harr et al., 2016), aligned to the mouse GRCm38/mm10 reference sequence. The fragment shown represents the one used for the promoter studies - only replacements with respect to the reference are marked, the two relevant regions discussed in the text are enlarged with their respective sequences. DOM represents *M. m. domesticus* populations, MUS represents *M. m. musculus* populations, CAS represents a *M. m. castaneus* population. T/A is the only mutation that correlates with the expression of the new promoter. Note that the transcriptional start site, marked as “0”, is located between the annotated site for this transcript, which would be 31 bp further upstream, and the site from which the bulk of the transcripts generated in the RNASeq experiment starts, which is 8 bp downstream (see Supplementary Figure S2 for a corresponding sequence depiction of this region). The bottom shows the scheme of the four constructs tested in cell culture and their expression levels measured as relative luminescence units [RLU] (see Methods). Error bars indicate standard deviations from eight replicates; all differences are significant (*p* < 0.05, *t*-test).

To further understand the *cis*-regulation of the spermatid specific 1.6 kb Map2k7α1 transcript, we analyzed genomic sequences in a 500 bp window upstream of the presumptive transcription start site in different wild populations, subspecies, and species of *M. m. domesticus, M. m. musculus, M. m. castaneus, M. spretus and M. spicilegus* (Figure 4). Only one SNP at -84 bp (with respect to the start site of transcription at chr8:4,240,136—see legend Figure 4 and Supplementary Figure S2 for alternative start sites) is correlated with the expression of the spermatid specific isoform in the different populations. An adenine is found in populations that show an expression of the new transcript, whereas thymine is present in those without expression (Figure 4).

Empirical data provide evidence that specific transcription activity in reproductive tissues and particularly in spermatogenesis is regulated by very short proximal promoters (Zambrowicz et al., 1993; Li et al., 1998; Reddi et al., 1999; Blaise et al., 2001; Topaloglu et al., 2001; Han et al., 2004; Scieglinska et al., 2004; Somboonthum et al., 2005; Acharya et al., 2006). Those studies demonstrated that proximal promoters shorter than 300 bp, or even less than 100 bp, are sufficient to drive spermatid-specific expression in mice. Additionally, it was shown that a 5′-CACACA motif ∼170 bp upstream of the transcription start serves as an insulator in the spermatid specific expression of the SP-10 gene (acrosomal vesicle protein 1—Acrv1), and it was suggested that insulators might generally play an important role in maintaining spermatid specific transcription (Reddi et al., 2003; Acharya et al., 2006; Abhyankar et al., 2007; Reddi et al., 2007).

Such a 5′- CACACA motif can be found at around -259 base pairs upstream the transcription start site of the testis-specific Map2k7-α1 RNA (Figure 4). These considerations raise the question, whether a short sequence carrying the −84 A mutation in combination with the -259 5′- CACACA motif would meet the requirements to serve as a testis specific promoter. Therefore, we tested a fragment representing the genomic sequence between −487/+43 (and thus larger than the expected size of the promoter region) of the new *Map2k7* testis promoter in cell culture-based luciferase expression assays (Figure 4).

The expression of most interest in this context is restricted to late spermatids. Culturing these types of cells is very difficult due to its haploid post-meiotic stage with condensed chromatin. A well-established spermatid cell culture model is not available, and alternative cell lines have the disadvantage that they will most likely not recognize the spermatid-specific *Map2k7* promoter. Therefore, we have chosen the widely used NIH/3T3 fibroblast cell line in the absence of better options for this experiment.

It cannot be expected that the tested fragment is sufficient to drive luciferase expression in non-spermatid cells, but the expression level can likely be raised by deleting the 5′-CACACA motif at −259, if the assumption is correct, that this sequence maintains spermatid specific transcription by acting as an insulator in other cells. Thus, −487/+43 fragments lacking the 5′-CACACA motif at −259 were generated as well. It can be assumed that the −487/+43 fragments do not contain enhancer elements that promote expression in fibroblasts. Therefore, a CMV enhancer was ligated upstream to both versions. All four constructs (wild type, wild type with deleted insulator, CMV enhancer + wild type, CMV enhancer + wild type with deleted insulator) were created as *M. m. domesticus* variants with an adenine at position −84 and as *M. m. musculus* variants with a thymine at position −84. We found that the *M. m. domesticus* variant generates significantly higher signals compared to *M. m. musculus* in every combination. The different replicates are consistent, indicated by relatively small standard deviations (Figure 4). Deletion of the 5′-CACACA motif at −259 indeed increases the expression strength in both variants. The presence of an enhancer potentiates the effects as expected. These data provide strong evidence that the adenine at position -84 enhances the activity of the basal promoter. For this reason, it can be supposed that a major contribution of this mutation to the expression difference between *M. m. domesticus* and *M. m. musculus* in late spermatids is likely. The sequence 5′-CACACA represses the action of an adjacent enhancer to a certain extent. This finding supports the hypothesis that it acts as an insulator in the spermatid-specific *Map2k7* promoter.

### Functional Analysis

To assess a possible functional role of the new spermatid specific *Map2k7* promoter, a knockout mouse was generated in which a fragment of 593 bp including the promoter was deleted in the *M. m. domesticus* background (see Methods and targeting strategy in Supplementary Figure S2). The knockout was designed in a way that it should not interfere with the conserved *Map2k7* transcript.

The knockout animals were fully viable and showed no overt phenotype. KO animals are, on average, a bit heavier but have lower normalized testis weights (Table 1). Given the specific expression of the new transcripts during a crucial phase of sperm maturation, we also assessed sperm motility phenotypes. KO animals have fewer motile sperm and fewer progressive sperm (Table 1). All differences are significant at *p* < 0.05 (*t*-test, 2 sided).

**TABLE 1:** Testis and sperm analysis for the Map2k7-α1 promotor KO animals versus wild type (WT) animals. Testis weights were normalized with mouse body weights. Sperm analysis was done with a CEROS Sperm Analyzer (Hamilton Thorne, Beverly MA) setup. Two-sided t-tests were done to calculate the *p*-values. SD is standard deviation (in italics).

Genotype		Mouse weight (g)	Normalized testis weight (g)	Motile sperm (%)	Progressive sperm (%)
WT (N = 10)	averages	25.14	0.20	48	26
	*SD*	*1.42*	*0.03*	*6*	*5*
KO (N = 18)	averages	27.59	0.17	42	22
	*SD*	*2.07*	*0.01*	*7*	*5*
	*p*-values	0.003	0.001	0.021	0.031

### RNASeq Analysis

A comparative RNASeq analysis with RNA from knockout mice versus wildtype mice was used to determine whether the 1.6 kb Map2k7-α1 testis-specific transcript influences the expression of other genes. The RNA was collected from three different tissues of the male reproductive organs, the testis, the caput epididymis, and the cauda, with eight biological replicates each. The testis is the place of the primary sperm production. The sperm from the testis move through the caput epididymis, where they mature and are eventually stored in the cauda. While the chromatin of post-meiotic sperm is condensed, there is still some transcriptome turnover (Ren et al., 2017), and the epididymal cells contribute to this transcriptome turnover as well (Shi et al., 2021).

**FIGURE 5:**
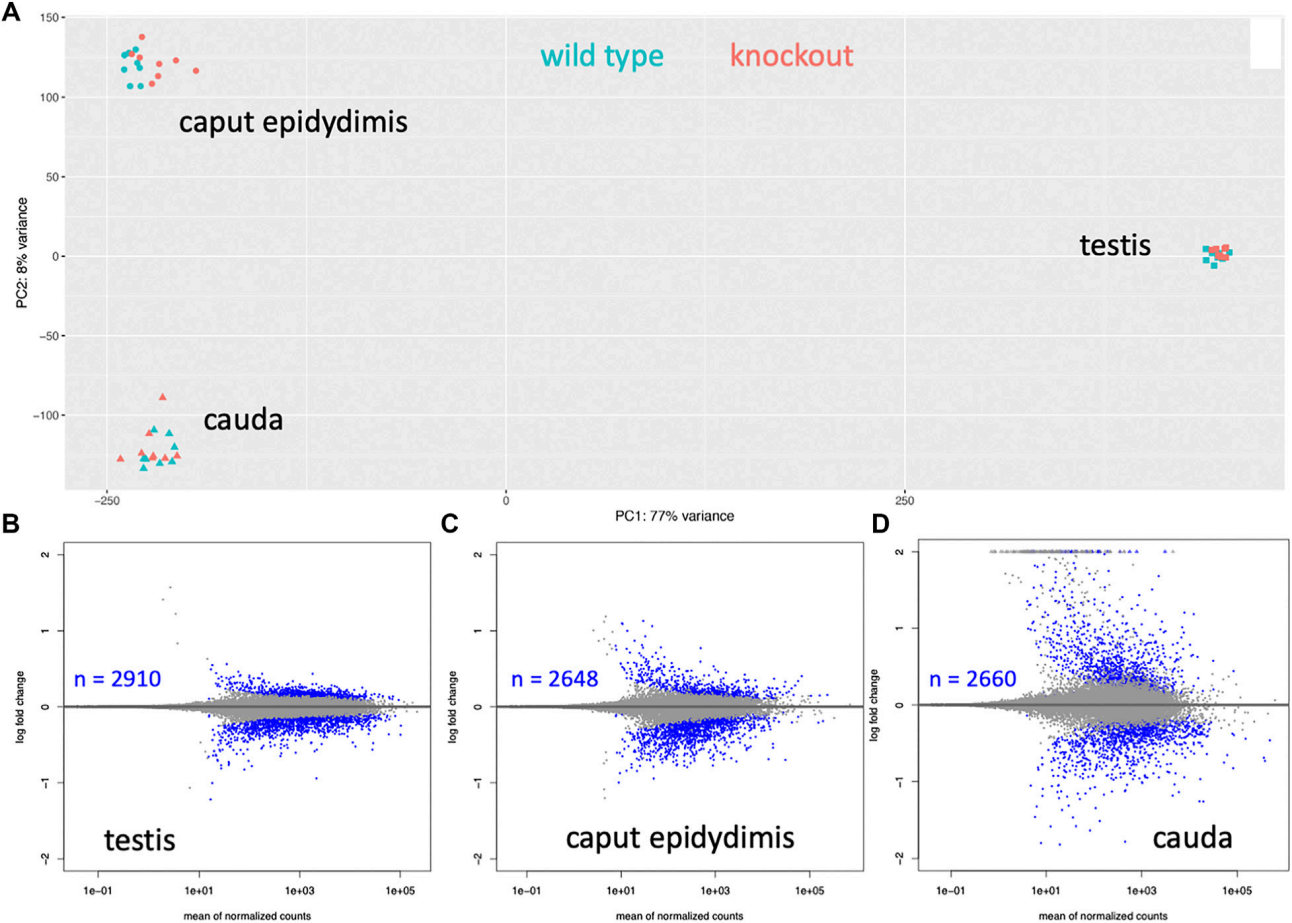
Whole transcriptomes analysis of three tissues from wild type versus knockout mice. **(A)** Overall PCA comparison. Strong differentiation is seen between the tissue samples, implying that those transcript sets are very different. The differences between wild type and knockouts are much smaller for each tissue. **(B–D)** Significantly differentially expressed genes. A dot represents each gene, genes with p_adj_ < 0.05 values are plotted as blue dots. The number of significantly differentially expressed genes is provided as an inset for each tissue.

The overall analysis of the transcriptome data in the PCA showed that the samples from each of the three tissues were very different, implying that there is indeed a major turnover of RNA between these stages, either due to differential stability, or to new transcription. On the other hand, differences between wild type and knockout are much smaller (Figure 5A). Still, since we used eight replicates for each tissue, we have a very high sensitivity to detect even small transcriptome changes (Xie et al., 2020). Accordingly, we find thousands of genes with significant expression differences (i.e., p_adj_ values < 0.05 in the DeSeq2 analysis), but mostly with relatively low log2fold-changes (Figures 5B–D). Interestingly, however, the cauda samples show a set of genes with very high positive log2fold changes (see set of dots on the top of the panel for cauda in Figure 5D).

First, we asked whether kinase signaling processes are specifically affected, which would suggest that the shortened protein of the Map2k7-α1 transcript could be involved in signaling, although lacking the D-domains that mediate the interaction with JNK (Tournier et al., 1999). had shown a residual kinase activity for this protein, but only under over-expression conditions in cell culture. However, the top biological process GO terms among the significant genes do not include “kinase signaling”, “signal transduction” or “Jnk cascade” in either of the data sets [based on a GO analysis with Panther (Mi et al., 2013)—see Supplementary Table S1]. Instead, the top GO terms indicate involvement in the meiotic division and chromosome segregation for testis, an involvement in extracellular matrix organization for the caput epididymis, and an involvement in peptide biosynthetic processes for the cauda (Supplementary Tables S1B,D,F). Hence, it is unlikely that the primary function of the Map2k7-α1 transcript is related to residual kinase signaling activity.

To get a further insight into the functional changes in the knockout animals, we focused on the genes that are most highly expressed in the respective tissues (based on the length-normalized baseMean counts of the RNASeq data), since even small concentration changes in such genes could have a marked influence on the phenotype.

For most of the highly expressed genes that we identified in the significant gene lists for testis and cauda epididymis, one can retrieve studies with functional information from knockout experiments in mice, and almost all of these find an effect on sperm maturation and/or sperm mobility (Table 2). Hence, while the relative expression changes are not large, the effect of the Map2k7-α1 transcript knockout may well be mediated via these genes. Interestingly, for the cauda, we found a rather different pattern. Most of the top expressed genes in the list are not specific for the cauda, but are more broadly expressed (e.g., an enzyme, actin, and a ribosomal protein). Interestingly, several code for immunity proteins, including sperm-specific antimicrobial peptides. Another major difference in the cauda transcriptome is a set of 37 genes with very high log2fold changes, i.e., expression at a much higher level in the knockout than in the wild type. Intriguingly, most of these are generally expressed motor proteins, and the role of such proteins in spermiogenesis has only recently been fully recognized (Wu et al., 2021).

**TABLE 2:** List of top significant genes in the RNASeq analysis for the three tissue samples. Named genes (i.e., predicted genes were not included) were retrieved from the DeSeq2 output ranked according to baseMean/mRNA length and expression characteristics (see Supplementary Table S1 for full results).

Tissue	Gene name	Description	log2 fold-change	Padj	Expression	Function	Literature
Testis (top 10 most highly expressed genes that are specifically expressed in testis)^a^							
	*Tnp2*	Nuclear transition protein 2	−0.37	0.0021	testis	conversion of nucleosomal chromatin in spermatids, involved in sperm motility	Adham et al. (2001)
	*Smcp*	Sperm mitochondrial-associated cysteine-rich protein	−0.25	0.0006	testis	involved in sperm motility and fertilization	Nayernia et al. (2002)
	*Gapdhs*	glyceraldehyde-3-phosphate dehydrogenase, spermatogenic	−0.12	0.0206	testis	required for normal sperm motility and male fertility	Huang et al. (2017)
	*Fhl4*	Four and a half LIM domains 4	0.09	0.0186	testis	may affect sperm maturation and morphology	Lardenois et al. (2009)
	*Tuba3a*	tubulin, alpha 3A, spermatogenic	−0.14	0.0027	testis	required for the production of normal spermatozoa	Akter et al. (2021)
	*Crisp2*	Cysteine-rich secretory protein 2	0.10	0.0444	testis	regulates calcium fluxes during sperm capacitation, essential for fertility	Curci et al. (2020)
	*Akap4*	A-kinase anchor protein 4	0.13	0.0037	testis	major structural component of sperm fibrous sheath, plays a role in sperm motility	Zhang et al. (2019)
	*Tuba3b*	tubulin, alpha 3B, spermatogenic	−0.11	0.0036	testis	required for the production of normal spermatozoa	Akter et al. (2021)
	*Cox8c*	cytochrome c oxidase subunit 8C	−0.26	0.0000	testis	required for the production of normal spermatozoa	Akter et al. (2021)
	*H1fnt*	testis specific H1.7 linker histone	−0.15	0.0013	testis	required for cell restructuring and DNA condensation during spermiogenesis	Martianov et al. (2005)
Caput epididymis (top 10 most highly expressed genes that are specifically expressed in testis/epididymis)^b^							
	*Lcn8*	Epididymal-specific lipocalin-8	0.15	0.0493	epididymis	involved in sperm maturation and motility	Wen et al. (2021)
	*Lcn9*	lipocalin 9	0.14	0.0450	epididymis	may act redundantly to Lcn8	Wen et al. (2021)
	*Epp13*	Epididymal protein 13	-0.13	0.0378	epididymis	no fecundity defect in knockout mice	Noda et al. (2019)
	*Cst8*	cystatin 8 (cystatin-related epididymal spermatogenic)	0.24	0.0016	epididymis	capacitation of spermatozoa	Chau and Cornwall (2011)
	*Rnase10*	Ribonuclease-like protein 10	0.40	0.0000	epididymis	required for post-testicular sperm maturation	Krutskikh et al. (2012)
	*Adam7*	Disintegrin and metalloproteinase domain-containing protein 7	0.15	0.0108	epididymis	required for epididymal integrity, sperm morphology and motility	Choi et al. (2015)
	*Teddm1a*	Transmembrane epididymal family member 1a	0.28	0.0035	epididymis	no information on sperm effects	
	*Teddm2*	Transmembrane epididymal family member 2	0.34	0.0001	epididymis	no information on sperm effects	
	*Spink12*	serine peptidase inhibitor, Kazal type 12	−0.31	0.0003	epididymis	part of Spink family with redundant functions in sperm maturation	Jeong et al. (2019)
	*Defb12*	defensin beta 12	0.20	0.0033	epididymis	immunity protein	
Cauda (top 10 most highly expressed genes)^c^							
	*Crisp1*	Cysteine-rich secretory protein 1	−0.46	0.0013	testis, epididymis	required to optimize sperm flagellum waveform	Gaikwad et al. (2021)
	*Cd52*	CAMPATH-1 antigen	−0.69	0.0005	broad	immunity protein	Yamaguchi et al. (2008)
	*Gpx3*	Glutathione peroxidase 3	−0.73	0.0043	broad	protects cells from oxidative damage, involved in the maturation of sperm cells	Noblanc et al. (2011)
	*Defb28*	Defensin beta 28	−0.37	0.0404	testis, epididymis	immunity protein	
	*Spink8*	Serine protease inhibitor Kazal-type 8	−0.44	0.0009	testis, epididymis	part of Spink family with redundant functions in sperm maturation	Jeong et al. (2019)
	*B2m*	Beta-2-microglobulin	−0.39	0.0035	broad	component of the class I major histocompatibility complex (MHC)	
	*Wfdc15b*	WAP four-disulfide core domain protein 15B	−0.75	0.0002	testis, epididymis	Wfdc family members act in innate immune responses during epididymitis	Andrade et al. (2021)
	*Defb2*	defensin beta 2	−0.38	0.0336	testis, epididymis	immunity protein	
	*Acta2*	Actin, aortic smooth muscle	0.44	0.0409	broad	cell motility, may affect contractability of seminiferous tubules	Uchida et al. (2020)
	*Rplp1*	ribosomal protein, large, P1	−0.54	0.0110	broad	ribosomal protein	
Cauda (top 10 genes with highest log2fold changes)^c^							
	*Pvalb*	parvalbumin	8.93	0.0158	broad	no information on sperm effects	
	*Myl2*	myosin, light polypeptide 2, regulatory, cardiac, slow	8.36	0.0338	broad	motorprotein involved in spermiogenesis	Wu et al. (2021)
	*Myh7*	myosin, heavy polypeptide 7, cardiac muscle, beta	8.04	0.0405	broad	motorprotein involved in spermiogenesis	Wu et al. (2021)
	*Myl1*	myosin, light polypeptide 1	7.26	0.0170	broad	motorprotein involved in spermiogenesis	Wu et al. (2021)
	*Atp2a1*	ATPase, Ca++ transporting, cardiac muscle, fast twitch 1	7.05	0.0008	broad	Ca-gradient regulation, male fertility	Prasad et al. (2004)
	*Myh1*	myosin, heavy polypeptide 1, skeletal muscle, adult	6.73	0.0404	broad	motorprotein involved in spermiogenesis	Wu et al. (2021)
	*Actn2*	actinin alpha 2	6.40	0.0380	broad	motorprotein involved in spermiogenesis	Wu et al. (2021)
	*Sln*	sarcolipin	6.01	0.0307	broad	no information on sperm effects	
	*Tnnt3*	troponin T3, skeletal, fast	5.86	0.0050	broad	motorprotein involved in spermiogenesis	Wu et al. (2021)
	*Mylk4*	myosin light chain kinase family, member 4	5.85	0.0138	broad	motorprotein involved in spermiogenesis	Wu et al. (2021)

a Selected from the top 17 genes in the whole list (see Supplementary Table S1B).

b Selected from the top 27 genes in the whole list (see Supplementary Table S1D).

c Represent the top 10 genes from the list (see Supplementary Table S1F).

## Discussion

Based on comparative genomic and functional analysis, we show here that a new intra-intronic promoter has arisen in the mouse lineage 2–6 million years ago and has led to the evolution of a functionally new transcript within an otherwise highly conserved gene. The transcript is specific to the testis, and knockout combined with transcriptome analysis shows that it is functionally involved in sperm maturation. Interestingly, the new promoter activity also got secondarily lost in some mouse lineages, apparently due to acquiring a disabling mutation in the promoter region.

The emergence of evolutionary novelties out of regulatory changes is by now well documented in many species [see (Tautz, 2000; Wray, 2007; Carroll, 2008; Romero et al., 2012; Osada et al., 2017; Signor and Nuzhdin, 2018; Mattioli et al., 2020; He et al., 2021) for a subset of relevant papers and reviews]. In fact, it is so abundant that it often constitutes the first measurable differences in population and species divergence, including diverging mouse populations (Bryk et al., 2013), which raises the question of whether much of is initially neutrally evolving or could be functional (Fay and Wittkopp, 2008; Staubach et al., 2010; Hodgins-Davis et al., 2019; Hill et al., 2021). Given the evolutionary volatility of the Map2k7-α1 transcript, with its fast secondary loss after its initial emergence, one would typically have considered it to be mostly neutral and therefore subject to random fixation or loss. However, our data show that it has a clear functional role in spermatogenesis. Therefore, the high evolutionary dynamics of this transcript is more likely explained by the general effects of sexual selection that would be particularly effective in the germline and the gonads (Kleene, 2005).

There are several possibilities of how the Map2k7-α1 transcript could function. The first is that it leads to translating a truncated protein that codes only for *Map2k7* kinase but does not bind specifically to JNK. It could therefore phosphorylate other signaling proteins but in an unspecific manner. This would likely be detrimental rather than advantageous for the cells. Also, since we do not find GO terms that relate to signaling processes in the transcriptome analysis of knockout mice, we assume that the truncated protein is not expressed, or at least, not functional.

The second possibility is that Map2k7-α1 acts as a non-coding RNA. There are multiple ways non-coding RNAs can regulate other genes or gene complexes (Gil and Ulitsky, 2020; Statello et al., 2021), and some have been implicated in male infertility (Joshi and Rajender, 2020). In a previous study, we identified a testis-specific new transcript that has also emerged via a new promoter acquisition and for which we could infer that it acts as lncRNA in spermiogenesis (Heinen et al., 2009). However, given that most of the Map2k7-α1 RNA overlaps with the functional *Map2k7* transcripts, it would seem unlikely that it could have assumed such a function as non-coding RNA as a whole since most of its RNA is actually potentially coding. Only the new exon that emerged out of intronic sequences might have such a function.

On the other hand, the first AUG in this new transcript is embedded in an optimal Kozak-consensus-sequence (Kozak, 1987), and one would therefore expect that it leads to the translation of a 50aa ORF. The resulting peptide does not match with any other protein or domain in the data bases, since it is actually produced out of an alternative reading frame of the *Map2k7* gene. A structure prediction analysis is therefore currently not possible, because even the most refined algorithms require appropriate comparative data from other species (Diwan et al., 2021). This is generally a problem for all *de novo* emerged proteins, although comparative analysis of large numbers of them has suggested that they more likely have a higher intrinsic disorder, which makes them better tolerated in cells (Tretyachenko et al., 2017; Wilson et al., 2017; Fajardo and Tautz, 2021; James et al., 2021). Still, for at least one *de novo* emerged protein foldability has been experimentally confirmed (Bungard et al., 2017).

While it has long been thought that proteins that emerge out of such more or less random sequences would not be functional, it has by now become clear that the *de novo* evolution of proteins is well possible (Tautz and Domazet-Loso, 2011; Van Oss and Carvunis, 2019; Zhang et al., 2019). In fact, we have recently described such a case of a very recent *de novo* emergence of a protein that regulates pregnancy cycles in mice (Xie et al., 2019). In that case, we could prove the protein’s direct function in a knockout mouse that carried only a frameshift mutation in the protein. In the current study, we deleted the whole transcript, implying that it is not fully proven that it is the translated peptide that conveys the function. But studies with random peptide sequences in *E. coli* and plants have shown that a substantial fraction of them can directly affect their hosts (Bao et al., 2017; Neme et al., 2017; Fajardo and Tautz, 2021). Therefore, it seems possible that the new peptide encoded by the Map2k7-α1 transcript is indeed a *de novo* protein with a function. Hence, this would be a case where a pre-existing potentially functional sequence was “waiting” for a promoter emergence to allow it to become functional via overprinting of an existing reading frame (Rancurel et al., 2009; Neme and Tautz, 2013; Carter, 2021).

The ORF is actually already present in the outgroup species but would not be expected to be translated in these species. The fact that it got secondarily lost in the *M. m. musculus* subspecies is in line with the observation of fast gain and loss cycles of *de novo* evolved transcripts and proteins (Carvunis et al., 2012; Neme and Tautz, 2014; Palmieri et al., 2014). Nevertheless, our functional data show that the new transcript (and/or peptide) can still be functional despite this evolutionary instability.

## Data Availability

The datasets presented in this study can be found in online repositories. The names of the repository/repositories and accession number(s) can be found below: European Nucleotide Archive (accession: PRJEB39625).

## Author’s Note

Part of this work was done in the framework of the PhD thesis of the first author (Heinen, 2008).
