# Supplementary material for: Evolution of a New Testis-Specific Functional Promoter Within the Highly Conserved Map2k7 Gene of the Mouse
Source: Front Genet. 2022 Jan 5;12:812139. doi: 10.3389/fgene.2021.812139 (PMC8766832; doi:10.3389/fgene.2021.812139)
Supplement: Supplementary file 1 [file DataSheet2.docx]

Supplementary Material

**Supplementary file S1**

Exon specific Northen-Blotting and qPCR of *Map2k7*


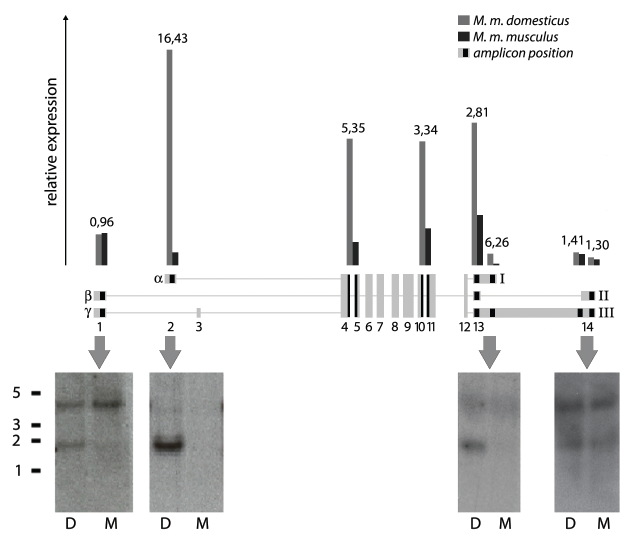


Exon specific *Map2k7* Northern blots and qRT-PCRs comparing *M. m. musculus* and *M. m. domesticus*. Testis RNA from *M. m. domesticus* (D) and *M. m. musculus* (M) was hybridized with different probes against certain parts of *Map2k7*. Probe positions of the respective blots are indicated with grey arrows. The size standard on the left displays kb. Relative expression of different qRT-PCR amplicons (about 100 bp in size) is shown at the top. Two amplicons span neighboring exons (4-5; 10-11). Positions of the different amplicons are indicated by black areas in the exon map. The respective expression values are displayed as bars above. The numbers on top of the bars represent the ratio of the domesticus to the musculus value. The results lead to the assumption of a strong prevalence of Map2k7-α1 in *M. m. domesticus* which is missing in *M. m. musculus*.

**Supplementary file S2**

Targeting strategy for Map2k7-α1 knock out:


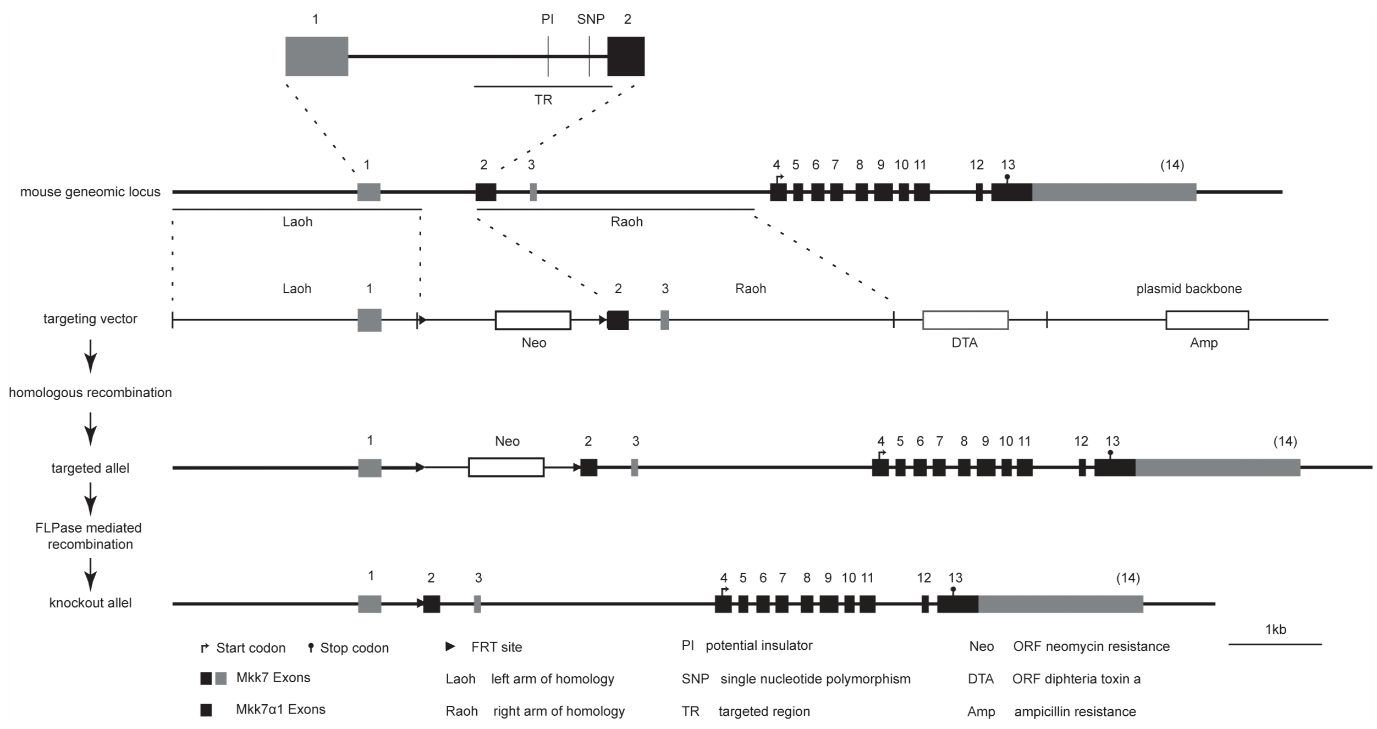


**Wildtype sequence**

chr8:4,239,310- 4,240,429 in GRCm38/mm10

gtcacctgcaccttgcaggctttcagactcgaagctacgccgctgtgactacaaccaagtcttttaactctgcaa

acagttatatctcttctgattcagtggttccactcctgccgagtcagtgactgtcaagaggtcccctcccctagc

agacagtccacacacgtgcatgcctatctgcccatgtaggtcactaagtcctcatctacatccgttttgattgga

|>replaced in knockout

ggcttctatttgacttctttggtcatatcagatggctcc*ttctaagcttggaaggaccttgtcactggacccag

>promotor test fragment

ctcactgcctcctacatacaggggcacctcatatctctaa#tgaccaactacttttcactattgctgtctagccc

tcaggaaacacatagccatctctcagcctggcagcctttgtctacagggctcaagtgactgctactactacagac

acctgtaaccagtgtagtcccatcgagtagaaacgcacctcctttctgagcctactgtctttgggcctgcctcct

-259

gacatacggtcctgtaccaaaagtgcttcctcttcctttctagacAcacatccagcttgtccaccatgaccacag

ttagcatccttgttgtatcccagatacccctgtcccaagttgtctttgctgaaagaatctggctttttttctccc

-84

ctctctgtccaacccttcctctgtccctcttgattcagcagaatgtcttctttatatcctctgtgatgtAatctt

L

tggagtatacatactatagttgtctgtgtggtcactatggtaagaggGgaaaggcagcctcctgtaggtgaaaat

0 B <| <

tctGttcactaCctggccacctggcctgactgac*cttcacagctt#gatcatcttcctgaagaggcattcagga

ttccctccatccctaccccttctggacaaagtcttccacgtttccttcctgggagtttcttccaggaactggaga

intron donor site

tacccagag>gtggggatgcatttcactgattctgcctgggaccagaggttgggcccctgctggattccagggcc

atccctccacggccctgtggatgagacagggttgggaatagggttctaggtgccataagagctgcctgtccctag

**Legend**: "0" marks the position that is used as reference for the transcription start site in Figure 4, "B" marks the start of the bulk of the transcripts detected in the RNA-Seq experiments, "L" marks the start of the longest annotated transcript in GRCm38/mm10.

The region replaced in the knockout construct is indicated by * and red labels, the region used for promotor analysis is indicated by # and blue labels.

Inserted NEO vector

gtcacctgcaccttgcaggctttcagactcgaagctacgccgctgtgactacaaccaagtcttttaactctgcaaacagttatatctcttctgattcagtggttccactcctgccgagtcagtgactgtcaagaggtcccctcccctagcagacagtccacacacgtgcatgcctatctgcccatgtaggtcactaagtcctcatctacatccgttttgattggaggcttctatttgacttctttggtcatatcagatggctcc*caattggataagcttgatatcgaattccgaagttcctattctctagaaagtataggaacttcaggtctgaagaggagtttacgtccagccaagctagcttggctgcaggtcgtcgaaattctaccgggtaggggaggcgcttttcccaaggcagtctggagcatgcgctttagcagccccgctgggcacttggcgctacacaagtggcctctggcctcgcacacattccacatccaccggtaggcgccaaccggctccgttctttggtggccccttcgcgccaccttctactcctcccctagtcaggaagttcccccccgccccgcagctcgcgtcgtgcaggacgtgacaaatggaagtagcacgtctcactagtctcgtgcagatggacagcaccgctgagcaatggaagcgggtaggcctttggggcagcggccaatagcagctttgctccttcgctttctgggctcagaggctgggaaggggtgggtccgggggcgggctcaggggcgggctcaggggcggggcgggcgcccgaaggtcctccggaggcccggcattctgcacgcttcaaaagcgcacgtctgccgcgctgttctcctcttcctcatctccgggcctttcgacctgcagcctgttgacaattaatcatcggcatagtatatcggcatagtataatacgacaaggtgaggaactaaaccatgggatcggccattgaacaagatggattgcacgcaggttctccggccgcttgggtggagaggctattcggctatgactgggcacaacagacaatcggctgctctgatgccgccgtgttccggctgtcagcgcaggggcgcccggttctttttgtcaagaccgacctgtccggtgccctgaatgaactgcaggacgaggcagcgcggctatcgtggctggccacgacgggcgttccttgcgcagctgtgctcgacgttgtcactgaagcgggaagggactggctgctattgggcgaagtgccggggcaggatctcctgtcatctcaccttgctcctgccgagaaagtatccatcatggctgatgcaatgcggcggctgcatacgcttgatccggctacctgcccattcgaccaccaagcgaaacatcgcatcgagcgagcacgtactcggatggaagccggtcttgtcgatcaggatgatctggacgaagagcatcaggggctcgcgccagccgaactgttcgccaggctcaaggcgcgcatgcccgacggcgatgatctcgtcgtgacccatggcgatgcctgcttgccgaatatcatggtggaaaatggccgcttttctggattcatcgactgtggccggctgggtgtggcggaccgctatcaggacatagcgttggctacccgtgatattgctgaagagcttggcggcgaatgggctgaccgcttcctcgtgctttacggtatcgccgctcccgattcgcagcgcatcgccttctatcgccttcttgacgagttcttctgaggggatcaattctctagagctcgctgatcagcctcgactgtgccttctagttgccagccatctgttgtttgcccctcccccgtgccttccttgaccctggaaggtgccactcccactgtcctttcctaataaaatgaggaaattgcatcgcattgtctgagtaggtgtcattctattctggggggtggggtggggcaggacagcaagggggaggattgggaagacaatagcaggcatgctggggatgcggtgggctctatggcttctgaggcggaaagaaccagctggggctcgactagagcttgcggaacccttcgaagttcctattctctagaaagtataggaacttcatcagtcaggtacataatatagatct*cttcacagcttgatcatcttcctgaagaggcattcaggattccctccatccctaccccttctggacaaagtcttccacgtttccttcctgggagtttcttccaggaactggagatacccagag>gtggggatgcatttcactgattctgcctgggaccagaggttgggcccctgctggattccagggccatccctccacggccctgtggatgagacagggttgggaatagggttctaggtgccataagagctgcctgtccctagagcagagtaagacctggtaaggctagtggctggcaggccccaaggagtcccctgc

Sequence after FRT recombination

gtcacctgcaccttgcaggctttcagactcgaagctacgccgctgtgactacaaccaagtcttttaactctgcaaacagttatatctcttctgattcagtggttccactcctgccgagtcagtgactgtcaagaggtcccctcccctagcagacagtccacacacgtgcatgcctatctgcccatgtaggtcactaagtcctcatctacatccgttttgattggaggcttctatttgacttctttggtcatatcagatggctcc*caattggataagcttgatatcgaattccgaagttcctattctctagaaagtataggaacttcatcagtcaggtacataatatagatct*cttcacagcttgatcatcttcctgaagaggcattcaggattccctccatccctaccccttctggacaaagtcttccacgtttccttcctgggagtttcttccaggaactggagatacccagag>gtggggatgcatttcactgattctgcctgggaccagaggttgggcccctgctggattccagggccatccctccacggccctgtggatgagacagggttgggaatagggttctaggtgccataagagctgcctgtccctagagcagagtaagacctggtaaggctagtggctggcaggccccaaggagtcccctgc

vector derived sequences underlined

FRT sites in yellow
